# Strategies for tackling *Taenia solium* taeniosis/cysticercosis: A systematic review and comparison of transmission models, including an assessment of the wider Taeniidae family transmission models

**DOI:** 10.1371/journal.pntd.0007301

**Published:** 2019-04-10

**Authors:** Matthew A. Dixon, Uffe C. Braae, Peter Winskill, Martin Walker, Brecht Devleesschauwer, Sarah Gabriël, Maria-Gloria Basáñez

**Affiliations:** 1 London Centre for Neglected Tropical Disease Research (LCNTDR), Department of Infectious Disease Epidemiology, Faculty of Medicine, School of Public Health, Imperial College London, London, United Kingdom; 2 MRC Centre for Global Infectious Disease Analysis, Department of Infectious Disease Epidemiology, Faculty of Medicine, School of Public Health, Imperial College London, London, United Kingdom; 3 One Health Center for Zoonoses and Tropical Veterinary Medicine, Ross University School of Veterinary Medicine, St. Kitts, West Indies; 4 Department of Infectious Disease Epidemiology and Prevention, Statens Serum Institut, Copenhagen, Denmark; 5 London Centre for Neglected Tropical Disease Research (LCNTDR), Department of Pathobiology and Population Sciences, Royal Veterinary College, Hatfield, United Kingdom; 6 Department of Epidemiology and Public Health, Sciensano, Brussels, Belgium; 7 Department of Veterinary Public Health and Food Safety, Faculty of Veterinary Medicine, Ghent University, Salisburylaan 133, Merelbeke, Belgium; University of California Berkeley, UNITED STATES

## Abstract

**Background:**

The cestode *Taenia solium* causes the neglected (zoonotic) tropical disease cysticercosis, a leading cause of preventable epilepsy in endemic low and middle-income countries. Transmission models can inform current scaling-up of control efforts by helping to identify, validate and optimise control and elimination strategies as proposed by the World Health Organization (WHO).

**Methodology/Principal findings:**

A systematic literature search was conducted using the PRISMA approach to identify and compare existing *T*. *solium* transmission models, and related Taeniidae infection transmission models. In total, 28 modelling papers were identified, of which four modelled *T*. *solium* exclusively. Different modelling approaches for *T*. *solium* included deterministic, Reed-Frost, individual-based, decision-tree, and conceptual frameworks. Simulated interventions across models agreed on the importance of coverage for impactful effectiveness to be achieved.

Other Taeniidae infection transmission models comprised force-of-infection (FoI), population-based (mainly *Echinococcus granulosus*) and individual-based (mainly *E*. *multilocularis*) modelling approaches. Spatial structure has also been incorporated (*E*. *multilocularis* and *Taenia ovis*) in recognition of spatial aggregation of parasite eggs in the environment and movement of wild animal host populations.

**Conclusions/Significance:**

Gaps identified from examining the wider Taeniidae family models highlighted the potential role of FoI modelling to inform model parameterisation, as well as the need for spatial modelling and suitable structuring of interventions as key areas for future *T*. *solium* model development. We conclude that working with field partners to address data gaps and conducting cross-model validation with baseline and longitudinal data will be critical to building consensus-led and epidemiological setting-appropriate intervention strategies to help fulfil the WHO targets.

## Author summary

*Taenia solium* infection in humans (taeniosis and neurocysticercosis) and pigs (cysticercosis) presents a significant global public health and economic challenge. The World Health Organization has called for validated strategies and wider consensus on which strategies are suitable for different epidemiological settings to support successful *T*. *solium* control and elimination efforts. Transmission models can be used to inform these strategies. Therefore, a modelling review was undertaken to assess the current state and gaps relating to *T*. *solium* epidemiological modelling. The literature surrounding models for other Taeniidae family infections was also considered, identifying approaches to aid further development of existing *T*. *solium* models. A variety of different modelling approaches have been used for *T*. *solium* including differences in structural and parametric assumptions associated with *T*. *solium* transmission biology. Despite these differences, all models agreed on the importance of coverage on intervention effectiveness. Other Taeniidae family models highlighted the need for incorporating spatial structure when necessary to capture aggregation of transmission stages in the environment and movement of animal hosts.

## Introduction

Infection by the cestode *Taenia solium* contributes to a significant and underreported public health and economic burden in low and middle-income countries [1, 2]. A transmission cycle including humans and pigs is facilitated by the free-roaming behaviour of pigs in subsistence and minimal biosecurity farming environments [3, 4]. Humans become definitive hosts when consumption of raw or undercooked cyst-infected pork leads to the tapeworm infection taeniasis (henceforth referred as taeniosis as per Kassai et al. [5]). Humans can also act as accidental intermediate hosts when *T*. *solium* eggs are ingested. In this instance, migration of the larval stage of *T*. *solium* to the central nervous system can result in neurocysticercosis (NCC) [6]. Human cysticercosis especially occurs in high-risk settings where poor hygiene and sanitation standards prevail [7, 8].

NCC is associated with epilepsy and a recent review found that 31.5% of epilepsy cases could be due to NCC in endemic settings [9]. The Foodborne Disease Burden Epidemiology Reference Group (FERG) under the World Health Organization (WHO) estimated that NCC-associated epilepsy accounted for approximately 2.8 million disability-adjusted life years (DALYs) globally in 2010, concluding that NCC contributed the largest number of DALYs in a list of priority foodborne parasites [10]. In addition to its impact on public health, *T*. *solium* infection in pigs is associated with a substantial economic burden due to the decreased market value of infected pigs [11, 12] and market distortion resulting from farmers adopting informal avenues for selling infected meat and animals [13, 14].

Combatting the burden associated with *T*. *solium* infection was initially recognised in the WHO “Global Plan to Combat Neglected Tropical Diseases (2008–2015)” [15] and by WHO Member States at the World Health Assembly [16]. More specifically, the 2012 WHO roadmap on neglected tropical diseases (NTDs) [17] set out the goal of scaling up interventions for *T*. *solium* in selected countries by 2020. This target was predicated on having achieved, by 2015, the establishment of a validated strategy to meet such a goal. Despite the declaration by the WHO of being ‘tool ready’ for pig-, human-, and environment-orientated interventions [18], the effective implementation of such intervention tools in endemic settings will present considerable challenges. It is likely that interventions will need to be tailored to local epidemiological circumstances, local pig husbandry practices and socio-cultural behaviours [19]. Even with epidemiological setting-appropriate strategies identified, a framework for supporting and implementing needs to be present within a control strategy. Braae et al. have proposed such a framework towards the control and elimination of *T*. *solium* [20].

Infectious disease modelling can support *T*. *solium* control and elimination strategies by improving understanding of the key transmission dynamics processes that shape epidemiological patterns and by comparing, optimising, and estimating the cost-effectiveness of tailored strategies applicable for control in local settings [21,22]. Following the 2012 London Declaration on NTDs [23], an international collaboration of infectious disease modellers emerged under the umbrella of the NTD Modelling Consortium (https://www.ntdmodelling.org/) to provide modelling and quantitative support to address questions surrounding the feasibility of achieving the WHO 2020 call targets with current or alternative/complementary strategies. For example, outputs using multi-model comparisons and field data have improved knowledge of epidemiological processes, such as examining the feasibility of *Onchocerca volvulus* elimination in Western Africa [24], or cross-validation with epidemiological data has enabled consensus-based evidence to emerge, as seen with the development of alternative mass drug administration guidelines to target lymphatic filariasis elimination [25].

In order to develop a comprehensive research agenda towards formulation of cost-effective strategies for the control and elimination of *T*. *solium* taeniosis/cysticercosis in the context of the WHO NTD 2015/2020 call for *T*. *solium*, this article seeks to compare and identify gaps in existing *T*. *solium* transmission dynamics models. We follow the approach of Nouvellet et al. [26] and Pinsent et al. [27] who have synthesized and compared a wide range of models for Chagas disease and trachoma, respectively. By assessing the current state of the field, we highlight differences in structure of published models, sources of uncertainty and the data used to motivate, inform and parameterise such models. We compare the main conclusions drawn from each model and uncover knowledge gaps related to model complexities and data needs. In addition to a comparison of *T*. *solium* transmission models, we review models representing the other members of the Taeniidae family to consider where future development of existing *T*. *solium* models may be focussed. We hope this work will therefore form the basis for improved dialogue between field epidemiologists, programme managers, and modellers.

## Methods

### Search strategy

We conducted a systematic review of modelling studies to understand population dynamics or effects of interventions caused by members of the Cestoda: Taeniidae family (i.e. *Echinococcus*, *Taenia*). A systematic review, conducted by Atkinson et al. [28], had focussed on assessing *Echinococcus* models only and has been consulted to corroborate our findings in this review. We performed a search for eligible studies in PubMed, without date or language restrictions, in January 2018, using the search terms: (Taeni* OR Echino* OR Cesto* OR cysticerc* OR hydatid*) AND (model OR models OR modelling OR modeling OR simulat*) AND (dynamics OR transmission OR control).

### Selection criteria and assessment

**Fig 1:**
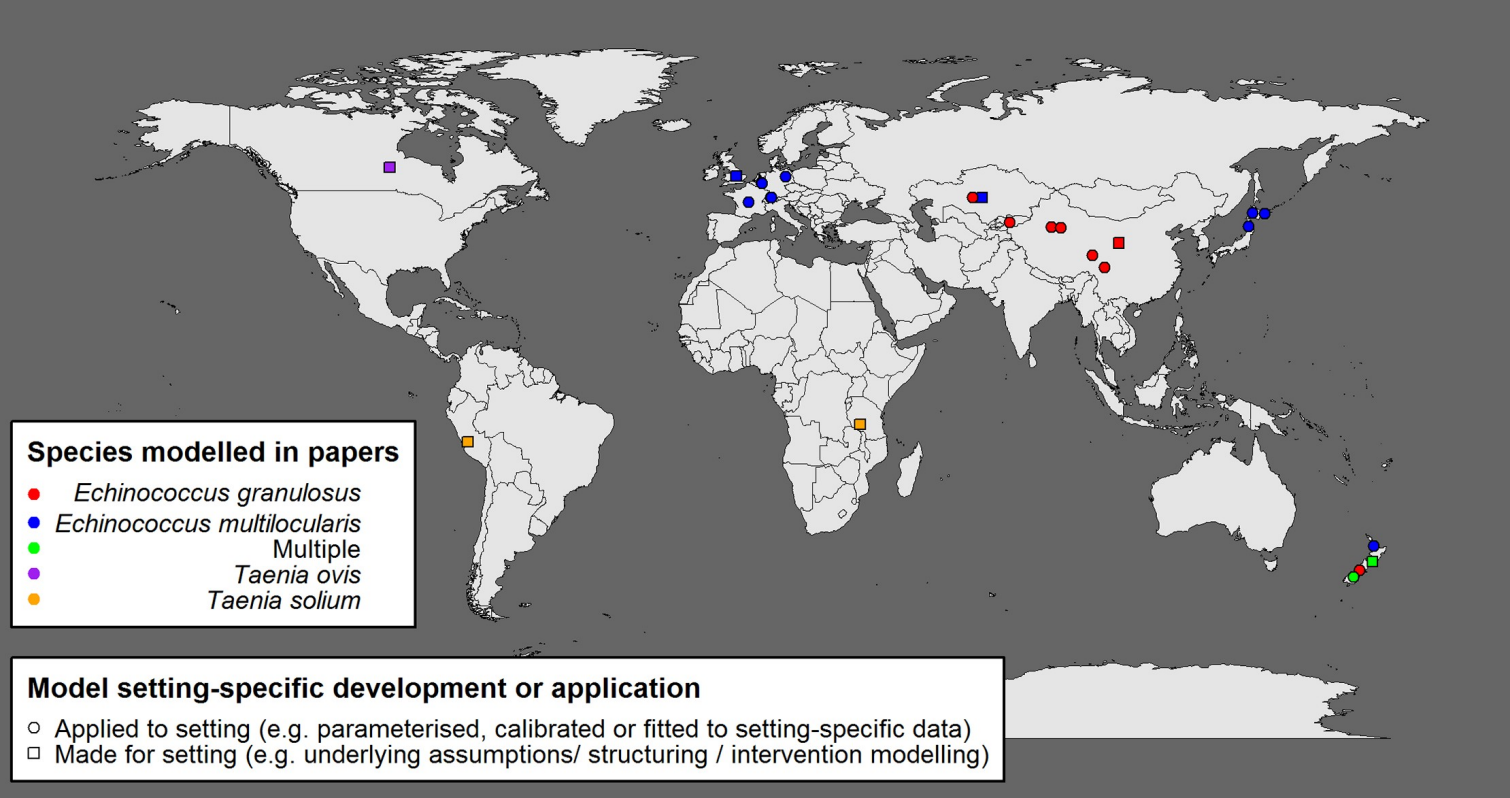
Geographical distribution of locations for which models have been developed or applied. Datapoints represent locations for model development, parameterisation and application, with colour related to species modelled and shape related to distinction between models developed for a specific setting compared to models applied to a setting (e.g. parameterisation, calibration). In most situations, models were applied to a country or local level (then approximate co-ordinates for centre of country or locale, e.g. district or city were applied for mapping). Those models not applied to specific country settings were therefore omitted (n = 4). The map has been created in the R package ‘maps’ using the base map.

The PubMed search output was reviewed by the following method: 1) title and abstracts were reviewed and articles were excluded if they were related to parasites or diseases different from those relating to the Taeniidae family; 2) all full texts were retrieved from those abstracts that met the inclusion criteria; 3) each article was reviewed for descriptions of mechanistic transmission models with specifications that addressed parasite prevalence, incidence, or intensity. Models addressing only spatial distribution or parasite abundance within a single host (i.e. not considering transmission between host species), or risk assessment models that did not consider explicitly transmission processes, were excluded. Literature found through the systematic search was supplemented by specific searches of references and papers known to the authors or cited in the papers obtained (Supplementary S1 Flow Diagram). Papers based on re-application or minimal modifications to the original models were excluded. Identified models were divided into groups based on model type and characteristics (Table 1). Geographic distribution of locations where models have been developed and applied are presented in Fig 1. This review is compliant with the PRISMA checklist for systematic reviews [29] and available in Supplementary S1 Table.

**Table 1:** Summary (in chronological order of publication) of the 28 models identified from the systematic search and included for analysis.

Model [Ref.]	Parasite species	Setting(s)	Type of model	Role of stochasticity
1) Harris et al. 1980 [30]	*Echinococcus granulosus*, *Taenia ovis*, *Taenia hydatigena*	New Zealand	Markov chain decision process	Deterministic
2) Roberts et al. 1986 [31]	*E*. *granulosus*	Australia / New Zealand	Force-of-infection (FoI) model (fitted to age-prevalence / age-abundance data) & integrodifferential equation model to determine equilibrium prevalence	Deterministic with stochastic elements
3) Roberts et al. 1987 [32]	*T*. *ovis*, *T*.* hydatigena*	Australia / New Zealand	As in Roberts et al. [30]	Deterministic with stochastic elements
4) Lawson et al. 1988 [33]	*E*. *granulosus*, *T*.* ovis*, *T*.* hydatigena*	New Zealand	Extending the integrodifferential equation model of Roberts et al. [30] to include infection control and economic assessment	Deterministic with stochastic elements
5) Roberts & Aubert, 1995 [34]	*Echinococcus multilocularis*	France	Compartmental, prevalence, population based	Deterministic
6) Gonzalez et al. 2002 [35]	*Taenia solium*	Peru	Decision tree	Stochastic
7) Torgerson, 2003 [36]	*E*. *granulosus*	China	FoI model^a^ with delay representing cyst maturation in intermediate host and simulating interventions	Deterministic
8) Hansen et al. 2003 [37]	*E*. *multilocularis*	Germany	Compartmental (“grid-based”) & individual based (spatially explicit)	Deterministic with stochastic elements
9) Ishikawa et al. 2003 [38]	*E*. *multilocularis*	Japan	Compartmental, population based	Deterministic
10) Milner-Gulland et al. 2004 [39]	*E*. *multilocularis*	Kazakhstan (arid/ semi- arid areas)	Spatially-explicit, coupled habitat-demographic model	Stochastic
11) Takumi & Van der Giessen, 2005 [40]	*E*. *multilocularis*	Netherlands / wider Europe	Compartmental, mean number, population based	Deterministic
12) Danson et al. 2006 [41]	*E*. *granulosus*, *E*.* multilocularis*	Non-specified	Conceptual model	N/A
13) Kyvsgaard et al. 2007 [42]	*T*. *solium*	Latin America (Bolivia, Peru, Mexico, Guatemala)	Reed-Frost (chain binomial model)	Deterministic with a stochastic version
14) Heinzmann & Torgerson, 2008 [43]	*E*. *granulosus*	Kazakhstan	FoI models^a^ extended to include age-truncated and age-dependent infection processes	Deterministic
15) Nishina & Ishikawa, 2008 [44]	*E*. *multilocularis*	Japan	Compartmental (population) and individual based	Deterministic with stochastic elements
16) Takumi et al. 2008 [45]	*E*. *multilocularis*	Netherlands	Compartmental, mean number of parasite stages, population based, spatially explicit	Deterministic
17) Torgerson et al. 2009 [46]	*E*. *granulosus*	Kyrgyzstan	FoI model^a^ extended to model variation in number of protoscolices per sheep	Deterministic
18) Kato et al. 2010 [47]	*E*. *multilocularis*	Japan	Compartmental, population based	Deterministic
19) Huang et al. 2011 [48]	*E*. *granulosus*	China	Individual based	Stochastic
20) Wang et al. 2013 [49]	*E*. *granulosus*	China	Compartmental, population based	Deterministic
21) Wu et al. 2013 [50]	*E*. *granulosus*	China	Compartmental, population based	Deterministic
22) DeWolf et al. 2013 [51]	*T*. *ovis*	Canada	Compartmental, spatially explicit	Deterministic
23) Lewis et al. 2014 [52]	*E*. *multilocularis*	Switzerland	FoI model^a^ exploring different functional forms for FoI and immunity	Deterministic
24) Braae et al. 2016 (cystiSim) [53]	*T*. *solium*	Tanzania	Individual based	Stochastic
25) Wang et al. 2017 [54]	*E*. *granulosus*	China	Compartmental, population based^b^	Deterministic
26) Otero-Abad et al. 2017 [55]	*E*. *multilocularis*	Switzerland	FoI model^a^ extended to include time-dependent and age-dependent infection pressure & spatial variability	Deterministic
27) Winskill et al. 2017 (EPICYST) [56]	*T*. *solium*	Sub-Saharan Africa	Compartmental, population based	Deterministic
28) Budgey et al. 2017 [57]	*E*. *multilocularis*	United Kingdom	Compartmental & individual based, spatially explicit	Deterministic with stochastic elements

FoI: Force of Infection

^a^ indicates modified modelling based on original Roberts et al. [31, 32]

frameworks

^b^ indicates model extension based on original modelling work by Wang et al. [49].

Identified studies were then analysed and data extracted based on the following headings: *Reference*, *Year*, *Title*, *Journal*, *Parasite genus*, *Parasite species*, *Motivation*, *Type of model (including further specificities of model type)*, *Nature of model (including whether the model represents or not the totality of the transmission cycle*, *i*.*e*. *Full transmission vs*. *partial model)*, *Role of stochasticity*, *Representation of population dynamics*, *Explicit representation of spatial transmission*, *Spatial design*, *Parameterisation/calibration for specific setting(s)*, *Hosts (states) represented*, *Explicit representation of Environment*, *Source of parameters*, *Major assumptions and model simplification(s)*, *Assessment of parametric uncertainty*, *Interventions modelled*, *Model validation (informal/formal)*, *and Main findings*. The full data extraction tool is available in Supplementary S1 File.

## Results & discussion

A systematic search of the literature yielded 23 papers plus two papers known to authors, and three identified through additional searches, for inclusion in the analysis. Of these, four studies modelled *T*. *solium* exclusively; 20 modelled infection by *Echinococcus* spp., one focussed solely on *Taenia ovis*, one on *T*. *ovis* and *Taenia hydatigena*, and the remaining two addressed *Echinococcus spp*. and *Taenia spp*. (other than *T*. *solium*) infections (*T*. *ovis* and *T*. *hydatigena*).

Results are first presented with an in-depth analysis of *T*. *solium* dynamic transmission models, followed by an assessment of the other Taeniidae family transmission models to identify possible modelling gaps and areas for future development of *T*. *solium* dynamic transmission models.

### *Taenia solium* transmission models

Analysis of the *T*. *solium* papers revealed four models that could be classified as dynamic transmission models (Table 2). Different modelling approaches are used to simulate *T*. *solium* transmission, including a decision tree/stochastic simulation approach in Gonzalez et al. [35]; deterministic and stochastic versions of a Reed-Frost model in Kyvsgaard et al. [42]–a chain binomial model whereby chains of infection are generated by the assumption that infection spreads between individuals in discrete units of time under the binomial probability distribution [58]; an individual-based, stochastic model, cystiSim [53]; and a population-based, deterministic model, EPICYST [56]. Representation of the *T*. *solium* life cycle is captured with varying degrees of complexity within each model. Similarities and differences between the four dynamic transmission models are subsequently compared based on *T*. *solium* life-cycle and transmission features.

**Table 2:** Summary of the structure and key features of Taenia solium transmission dynamics models identified from the systematic literature search.

Variables	Gonzalez et al. (2002) [35]		Kyvsgaard et al. (2007) [42]	Braae et al. 2016 (cystiSim) [53]		Winskill et al. 2017 (EPICYST) [56]
**Basic model structure & purpose**						
Representation of population dynamics	Decision tree/ stochastic		Reed-Frost	Individual-based		Population-based
Role of chance	Stochastic		Deterministic and stochastic	Stochastic		Deterministic
Motivation	Assess the effectiveness and cost-effectiveness of interventions		Assess intervention scenarios and estimate the basic reproduction number (*R*_0_)	Assess the effectiveness of interventions, including the probability of elimination		Assess the effectiveness of interventions and estimate the basic reproduction number (*R*_0_)
**Features included in the model and availability**						
Infection stages featured	HT, PCC, HCC	HT, PCC		HT, PCC	HT, HCC, PCC	
Way of representing infection in hosts	States for HT include immature, mature, and post-infection contamination; PCC states progress from immature to mature cysts, and (EITB) positivity. New cases of HCC are a function of a pre-set exposure level	States for HT and infected and recovered (+immune) pigs change over time through a binomial chain		HT individuals progress through maturation of immature tapeworms to harbouring infectious, mature tapeworms considering death of tapeworms. Individual pigs, once infected, progress to infectious pigs through cyst maturation	States for HT, HCC and humans infected with both taeniosis and cysticercosis are represented; the prevalence of PCC changes over each time-step	
Host population demographics	Pig population sub-model (birth, litter size, age/sex, mortality). Human host modelled as function of adult tapeworm status	Temporally stable (pig population)		Temporally stable (pig population demography based on data from Mbeya/Mbozi districts, Tanzania)	Temporally stable	
Heterogeneity in host infection	Not included	Not included		Human (age-dependent infection), pig (high/low burden)	Pig (high/low burden)	
Host immunity assumptions	Infected pigs develop life-long immunity after treatment. Antibody (EITB) positive modelled in pig states (maternal antibodies or following infection), but not indicative of protective immunity	Humans not susceptible to new infections while infected with a tapeworm. Infected pigs can recover and develop life-long immunity over 3 months.		Pigs not susceptible to infection for 3 months after treatment (default assumption but changeable if necessary)	Pigs not susceptible to infection for 3 months after treatment (default assumption but changeable if necessary)	
Representation of eggs in environment	Not explicit. Environmental contamination determined as a fixed delay in transmission reduction once a HT carrier is cleared of infection (dependent on climate/hygiene parameter).	Not modelled		Environmental contamination is a function of individuals with HT. Decay in egg viability in the environment is included	Compartment tracking number of eggs; egg production rate (input) & egg death rate (output)	
Exposure to eggs in environment	Not modelled explicitly (simulation assigns PCC disease status based on PCC prevalence)	Not directly modelled. PCC is modelled as a function of infected humans at a given time (‘probability of infection at contact’ parameter)		Heterogeneous exposure among pigs (direct transmission via coprophagia leads to high burden or indirect (environmental contamination) transmission leads to low burden infection). Contact is assumed to be random.	Density-dependent exposure (product of contact rate & probability of infection upon contact) for both pigs and humans. Set proportion of pigs develop high or low burden infections.	
Exposure to cysts in pork	Not modelled explicitly (simulation assigns HT disease status based on HT prevalence)	HT is modelled as function of infected pigs slaughtered at a given time (‘probability of infection at contact’ parameter		Pigs transmit infection to humans based on either high or low infection burden at different probabilities	Frequency-dependent exposure (product of contact rate & probability of infection upon contact with high- or low-cyst burden pigs)	
Other major assumptions	Infection rates same for all pigs (all pigs become infected in first 6 months of life).	Random contact between hosts, all pigs slaughtered and consumed in simulation; constant egg shedding rate from tapeworm		Humans can only harbour one tapeworm at a time, rate of decay in egg viability (onset from tapeworm death)	No excess mortality in HCC, negligible impact of egg consumption on egg numbers in environment. No prepatent period of adult worms	
Spatially explicit/ migration included	No: single location and no migration	No: single location and no migration		No: single location and no migration	No: single location and no migration	
Diagnostic uncertainty modelled?	No	No		No	No	
Model availability	Book chapter, code unavailable	Publication, code unavailable		Publication and code available (GitHub: https://github.com/brechtdv/cystiSim)	Publication and code available (GitHub: https://pwinskill.github.io/EPICYST/index.html)	

*R*_0_: Basic reproduction number, HCC: human cysticercosis, HT: human taeniosis, PCC: porcine cysticercosis, EITB: enzyme-linked immunoelectrotransfer blotting.

### Heterogeneity in transmission

Kyvsgaard et al. [42] incorporates compartments for human taeniosis and porcine cysticercosis but does not consider heterogeneity in host infection states, such as age dependency or infection burden. Equally Kyvsgaard et al. [42] assume a human to pig transmission probability of 0.01, without providing evidence to support this. While Gonzalez et al. [35] also omits any infection heterogeneity, more complexity is introduced as human states include those infected with maturating stages of the adult tapeworm, and infection and antibody presence in pig compartments. In addition, a pig-population stochastic sub-model is implemented to simulate population dynamics in the absence of infection [35]. The cystiSim model [53] features heterogeneity in both exposure and infection by modelling high (resulting from direct coprophagia) and low (resulting from indirect environmental exposure) burden infections in pigs, along with age-dependent human infection. The EPICYST model [56] assigns a proportion of the infected pig population into high or low burden states and incorporates different transmission mechanisms in the life cycle; a density-dependent process for pig and human exposure to eggs and a frequency-dependent process [59] for human exposure to cysts in pork.

### Environmental transmission

Both the Kyvsgaard et al. [42] and Gonzalez et al. [35] models do not explicitly model infection in the environment, although an ‘infection potential’, analogous to environmental contamination, is generated in Gonzalez et al. [35]. This is based on the number of adult tapeworms and humans in the ‘post-infection contamination’ stage, with the latter produced by a fixed-delay in transmission reduction, which can be varied depending on different climatic and hygienic conditions as specified by parameter inputs. The number of eggs in the environment is explicitly modelled in EPICYST [56], while cystiSim [53] defines environmental contamination as an attribute of previous tapeworm carriers with removal of eggs implemented using an exponential decay function based on environmental studies of *Taenia saginata* egg survival on pastures and expert opinion [60]. Considerable uncertainty surrounds the rate at which *T*. *solium* eggs decay in the environment, reflected in the use of egg survival studies from other Taeniidae species to inform parameterisation (Table 3). The sensitivity analysis conducted in EPICYST [56] of the model output (cumulative number of human cysticercosis cases) to model parameters, indicated egg death rate as a highly influential and uncertain parameter, highlighting the need for more research into *T*. *solium* egg environmental viability and whether heterogeneity exists between settings.

**Table 3:** Represented parameters, derived and nominal values for Taenia solium transmission dynamics models, outlining how parameters are represented, derived and their nominal values.

Parameter	Gonzalez et al. (2002) [35]	Kyvsgaard et al. (2007) [42]	Braae et al. 2016 (cystiSim) [53]	Winskill et al. 2017 (EPICYST) [56]
**Host demographic parameters**				
Pig birth rate	Poisson process	0.25 per 3 months (*pig death rate ensuring a stable population size)*	Function of number of pigs slaughtered (*to ensure stable population size)*	Set to net rate—0.083 per month
Pig death rate/ average age at slaughter	Daily mortality probability	0.25 per 3 months (rate of pig slaughter)	Average age at slaughter of 1 year and always before 36 months	0.083 per month (derived from average slaughter age: 1 year)
Human birth rate	Not modelled	Not modelled	Not modelled	Set to net rate—0.0015 per month
Human death rate	Not modelled	Not modelled	Not modelled	0.0015 per month (derived from average life expectancy of 54 years)
**Egg-specific parameters**				
Egg decay	Not modelled	Not modelled	Exponential decay with rate parameter of 0.268 per month based on *Taenia saginata* data [60]	2 per month (derived from average life expectancy of eggs in environment of 2 weeks) based on *T*. *saginata* [60] and *Echinococcus multilocularis* data [65]
Egg production rate	Not modelled	Not modelled	1,500,000 per month	960,000 per month (range of 640,000 to 1,800,000)
**Transmission parameters**				
Proportion of pigs with low/high burden	Not modelled	Not modelled	Function of direct (coprophagia) or indirect (environmental contamination) transmission probabilities	0.8 (therefore proportion with high burden is 0.2)
Average cyst maturation duration (PCC)	75 days	Not modelled	90 days	Not modelled
Average duration of larval infection (PCC) & subsequent protective immunity	Not modelled	1-year duration of larval stage (*derives pigs’ recovery rate and rate of becoming immune*) with lifelong immunity	0 (No natural recovery assumed, based on relatively short lifespan of pigs)	0 (No natural recovery assumed)
Treatment-induced immunity duration (infected pigs)	Not modelled	Assumed to be lifelong	3 months	3 months
Duration cysts remain viable after treatment	28 days	0 (No delay)	0 (No delay)	0 (No delay)
Rate of human pork meal procurement	Not modelled	Not modelled	Not modelled	0.5 per month (assumes average of 6 pork meals per year)
Average duration of larval infection (HCC)	Not modelled	Not modelled	Not modelled	3 years (*derives the HCC recovery rate*).
Average pre-patent period (adult *T*. *solium* tapeworm)	~ 3 months (90 days)	3 months	3 months	0 (no pre-patent period modelled)
Adult *T*. *solium* tapeworm lifespan	3 years	1 year (*derives human recovery rate*)	1 year	2 years (*derives human recovery rate*)
Minimum age of pork consumption	Not modelled	Not modelled	24 months	Not modelled
Probability of transmission from pig to human	Not modelled	0.0005 (any pig)	0.00011 (pigs with low burden); 0.00015 (pigs with high burden)	0.0084 (pigs with low cyst burden); 0.0147 (pigs with high cyst burden)

HCC: human cysticercosis, HT: human taeniosis, PCC: porcine cysticercosis.

### Host recovery and immunity

Several consistencies and differences emerge across the *T*. *solium* models in relation to assumptions on host immunity (Table 3). For example, there is no inclusion of human immunity for taeniosis across the models, although Kyvsgaard [42] indicate that “spontaneous elimination of the parasite” occurs without providing details. Natural recovery (in the absence of interventions) from porcine cysticercosis is only modelled in Kyvsgaard et al. [42], with pigs transferred to a recovered compartment given a certain probability, and subsequently develop assumed “life-long” immunity given short life-expectancies. A breeding sow would likely live much longer and outlive this period, although determining the contribution of these animals to transmission is unclear given this sub-population does not generally represent slaughtering stock. Equally, the presence of natural recovery from porcine cysticercosis is unclear, given that average pig life expectancy is low in many settings and needs further clarification from field data. Protective immunity is only included following treatment and recovery of infected pigs [61,62] in both cystiSim [53] and EPICYST [56] for a period of 3 months.

The pig host immune response is more directly modelled by Gonzalez et al. [35]. Firstly, if born to a serologically positive sow, pigs produce antibodies (modelled as being enzyme-linked immunoelectrotransfer blot [EITB] positive); it is assumed that these antibodies persist for a period of 8 months, although the pig may still acquire infection during this time (therefore being already EITB positive when infected). Secondly, pigs born to serologically negative sows become EITB positive with a delay of 15 days following larval infection with immature cysts. These infected pigs progress to infection with mature cysts after a delay of 75 days and remain EITB positive. Only (simulated) treatment clears infection as pigs move to and remain in the treated state, indicating that they are resistant to re-infection following treatment [63]. The modelling approach taken by Gonzalez et al. [35] also calls into question the need to include a “diagnostic layer” in the other *T*. *solium* transmission models to represent outcomes from serological data (both antibody and antigen in human and pig hosts) which may not directly equate to underlying true infection status in the hosts as performed in onchocerciasis [24] and Chagas disease modelling [64].

### Adult tapeworm biology and the basic reproduction number (R0)

Gonzalez [35] and cystiSim [53] model the maturation of the adult tapeworm, from infected humans (with taeniosis) harbouring immature and mature adult tapeworms, while EPICYST [56] considers only mature tapeworms (for the human taeniosis infected compartment) and ommiting the pre-patent period as this is assumed to be 5–10 weeks compared to a significantly longer human life expectancy duration. Kyvsgaard et al. [42] uses the prepatent period to set the time-step for the chains of infection. Across the Gonzalez [35], Kyvsgaard et al. [42] and cystiSim [53] models, the pre-patent period is defined as 3 months although this is based on data from other Taeniidae species including *T*. *saginata* and *Echinococcus multilocularis* (Table 3).

Further parameters related to the adult tapeworm life history also vary between the models including the egg production rate, which is identified as an influential and uncertain parameter in the EPICYST sensitivity analysis [56], and the assumed average life span of the adult tapeworm reflecting the limited data associated with the adult tapeworm dynamics (Table 3). This has a direct bearing on the estimated basic reproduction number (*R*_0_) of *T*. *solium* and accounts for some of the variability in the estimates of *R*_0_ between EPICYST [56] (*R*_0_ of 1.4, 95% credible Interval: 0.5–3.6) and the Kyvsgaard et al. [42] (*R*_0_ of 1.75) models. The *R*_*0*_ estimated in Kyvsgaard et al. [42] does not consider pig infections, with the calculation based on the summation of new infected humans over time, although there is no distinction between new human cases and those continually re-infected, and this definition erroneously produces units of time for *R*_*0*_. By contrast, *R*_*0*_ calculated from EPICYST [56] reflects the whole system of transmission among pigs, humans and the environment. Further noting that *T*. *solium* is not dioecious but it is hermaphrodite species, the classical *R*_*0*_ for helminths is strictly only compatible with an intensity-based modelling framework. The *R*_*0*_ however as estimated in EPICYST [56] still provides a useful and valid threshold quantity for comparison, given that *R*_*0*_ for *T*. *solium* has been estimated exclusively to date using so-called microparasitic prevalence modelling frameworks.

### Intervention modelling

Human-directed interventions are simulated in all four dynamic transmission models (Table 4). Mass drug treatment irrespective of infection status are simulated in Gonzalez et al. [34], Kyvsgaard et al. [42] and cystiSim [53], while EPICYST [56] currently models a hypothetical test-and-treat (T&T) intervention based on the possible future availability of a specific and sensitive point-of-care test for taeniosis, although current diagnostics lack either or both sensitivity and specificity [66, 67], or in the case of a highly specific coproantigen test [68], are not commercially available yet. For example, the human-directed intervention modelled in EPICYST [56] is a hypothetical approach based on the rES33 EITB for antibody detection [69,70], which has substantially lower specificity than that currently modelled and most intervention studies measuring human taeniosis use the coproantigen ELISA test [71,72]. Another potential limitation across models is that human treatment efficacy may be lower in field settings compared to currently assumed estimates. However, efficacy can be adjusted by the user in cystiSim [53] and EPICYST [56] with the available code, allowing adaptation of the model to a given treatment efficacy.

**Table 4:** Input variables, interventions and principal outcomes for Taenia solium transmission dynamics models: Intervention included and main model outcomes.

Variables	Gonzalez et al. (2002) [35]	Kyvsgaard et al. (2007) [42]	Braae et al. 2016 (cystiSim) [53]	Winskill et al. 2017 (EPICYST) [56]
**Interventions modelled & baseline calibration (in publications)**				
Baseline calibration / model initialisation	2,000 humans (exposed), prevalence of 3% (HT), 45% PCC	1,000 humans, 200 pigs; prevalence of 2% (HT), 20% (PCC)	Model calibrated and initialised to data from Mbeya/Mbozi in Tanzania	10,000 humans, 2,000 pigs; prevalence of (HT) = 2%, (PCC) = 20%, (HCC) = 7%
Pig-directed interventions	Mass drug administration (MDA)	MDA, vaccination	MDA, vaccination	MDA, vaccination
Human-directed interventions	MDA of HT	Test-and-treat (T&T) of HT (*hypothetical*), MDA of HT	MDA of HT	T&T of HT (*hypothetical*)
Behaviour change/ environment-directed interventions	Not modelled	Improved sanitation, husbandry, meat inspection and cooking practices	Not modelled	Improved sanitation, meat inspection and husbandry
Intervention heterogeneity	Coverage, treatment efficacy, intervals between rounds	Coverage, treatment efficacy	Targeting specific age groups, coverage, treatment efficacy, intervals between rounds	Coverage, treatment efficacy
**Main outcomes**				
Primary outcome	No. of interventions (rounds) until local parasite elimination, discounted benefit	Basic reproduction number (*R_0_*), post-intervention prevalence reduction, proportion of runs achieving elimination	Predicted probability of elimination & duration to elimination	HCC cases averted, Basic reproduction number (*R_0_*)
Impact of interventions	Success of interventions highly sensitive to coverage. Intervening in both humans and pigs reduce the number of intervention rounds required to achieve local elimination. Only one intervention (3x human MDA with 2x pig MDA rounds with 100% coverage/90- day intervals) resulted in discounted benefit greater than no intervention scenario	*R*_0_ for *T*. *solium* reduced to <1 following behavioural change/ environmental interventions but variable for pig-/human-directed interventions. Human T&T leads to most runs achieving elimination, followed by pig vaccination (single strategy)	Pig-directed interventions result in highest probability of and shortest time to elimination but dependent on high coverage and efficacy. Lower coverage of pig-focussed interventions compensated by combining with other interventions	Biomedical (pig-/human-directed) interventions highly effective (applied singularly) & more effective than behavioural/ environmental interventions. Sensitivity analysis shows that human- and pig-focussed interventions are more robust to coverage/efficacy changes compared to other interventions
Other epidemiological findings	Seasonality (factors not detailed) had a limited impact on infection dynamics over time	*R*_0_ for *T*. *solium* was estimated at 1.75 (no 95% confidence interval) at baseline	Stable dynamics achieved (validated against no-intervention dataset from Mbeya/Mbozi in Tanzania)	*R*_0_ for *T*. *solium* was estimated at 1.4 (95% credible Interval: 0.5–3.6) at baseline

HCC: human cysticercosis, HT: human taeniosis, PCC: porcine cysticercosis; *R*_0_: Basic reproduction number, T&T: Test & Treat—this is based on testing for taeniosis and only treating suspected taeniosis cases, MDA: Mass drug administration.

In the pig host, mass treatment (using oxfendazole) and/or vaccination (e.g., the TSOL18 vaccine [73]) are simulated in all models except Gonzalez et al. [35], where only pig mass treatment is simulated. Pig-directed interventions achieve high efficacy from field studies [74,75] and this is reflected in the models. For example, the treatment efficacy of oxfendazole is assumed to range from 90% in cystiSim [53] to 99% in EPICYST [56] to 100% in both Gonzalez et al. [35] and Kyvsgaard et al. [42]. Pig vaccination efficacy has also been assumed to be high, having been set to 100% in Kyvsgaard et al. [42], 99% with an adjustment to account for the fact that some piglets may become infectious before a full course of vaccine can be administered in EPICYST [56], and 90% in cystiSim [53], where vaccination was combined with treatment in all modelled scenarios. While cystiSim [53] and EPICYST [56] permit user-specified efficacy changes, cystiSim [53] has the added benefit of allowing for age-targeted interventions in both pigs and humans.

Coverage of human- and pig-targetted interventions is included as a parameter in all models, with coverage levels fixed at 90% Kyvsgaard et al. [42], but varied in the EPICYST [56] sensitivity analysis and across intervention scenarios for cystiSim [53] and Gonzalez et al. [35]. Behavioural and environmental focussed interventions have also been simulated using EPICYST [56] and Kyvsgaard et al. [42], including improved sanitation, husbandry, meat inspection and cooking practices, by modifying nominal values of certain transmission parameters. A key finding across the models is that human- and pig- targeted interventions are generally sensitive to coverage levels (Table 4), although these interventions are more robust to changes in coverage compared to behavioural and environmentally focussed interventions in the EPICYST sensitivity analysis [56]. One important quantity that could therefore be estimated is the minimum fraction of pigs to be vaccinated to achieve transmission interruption and infection elimination. Limitations with current modelling of interventions, especially on the effectiveness of human-directed intervention approaches and on the realistic, achievable coverage levels, emphasise the need to design intervention simulations in conjunction with research groups involved field intervention trials. Equally, simulations need to be compared with data collected during interventions implemented in the field. For example, it is planned that cystiSim [53] predictions will be compared with data collected in Zambia as part of the CYSTISTOP programme and used to update model inputs from longitudinal infection data and to inform parameters of interest including pig population turnover and actual coverage [76].

The existing models need to be tested to determine their ability to accurately model field-specific targeted interventions. For example, cystiSim was used to test targeted anthelmintic treatment in school-age children given the age-structure of the host human, replicating the approach taken in Braae et al [77]. Requirements to model other targeted interventions, such as the inclusion of spatially explicit structure to capture ring screening/treatment strategies, as applied in northern Peru [71], will need further consideration.

#### Assessing the broader Taeniid transmission models towards identifying advances for *T*. *solium* transmission modelling

The majority of modelling studies captured in the systematic search focussed on the *Echinococcus* genus (*n* = 20), other *Taenia* species (*n* = 20) and a mixture of these two (*n* = 2), providing a number of approaches that could be adopted to support further development of *T*. *solium* dynamic transmission models. Roberts et al. [31, 32] devised simple models to estimate infection pressure (force-of-infection, FoI) when fitted to *Echinococcus granulosus*, *T*. *ovis*, and *T*. *hydatigena* age-prevalence and age-abundance data in intermediate and definitive hosts. A key driver of this work was to understand the density-dependent constraints induced by acquired immunity, identified in the intermediate host in *T*. *ovis* and *T*. *hydatigena*, and inferred in the definitive host of *E*. *granulosus* (canids and other carnivores) from saturation of age-prevalence and age-abundance profiles. This model of acquired immunity enabled estimation of the FoI, and rates of acquisition and loss of immunity in the host to inform *E*. *granulosus* full dynamic transmission models of Torgerson [36] and Huang et al. [48].

### Age-infection heterogeneities and force-of-infection modelling

The FoI models of Roberts et al. [31,32] were also used to estimate the *R*_0_ of *E*. *granulosus*, *T*. *ovis*, and *T*. *hydatigena* and to determine the equilibrium steady-state. Similar FoI models could be fitted to *T*. *solium* age-prevalence data and, if available, age-abundance data from pigs and humans, as already performed in Ecuador and Zambia [78,79], but applied to a wide variety of epidemiological settings to support setting-specific model parameterisation. The egg to human and egg to pig transmission coefficients were identified in the EPICYST sensitivity analysis [56] and could therefore be informed through FoI estimation. This approach could also be used to investigate different assumptions on age-exposure patterns, for example by implementing age-dependent, age-truncated or dynamic FoI modifications to the FoI models [43, 52] and acquisition of immunity. Age-dependent infection is incorporated into human dynamics in cystiSim [53], however FoI modelling could help to inform further age-dependent infection processes in pig and human populations in cystiSim [53] and EPICYST [56]. For example, there is some evidence for specific age trends in taeniosis infection, with the highest prevalence’s found in younger age groups as identified in the Democratic Republic of Congo [80], Peru [81], and Guatemala [82], which could be a result of protective immunity in older individuals or age-specific meat consumption trends. Age-stratified taeniosis prevalence data could support FoI modelling to better understand the rate of recovery from taeniosis, identified as an influential and uncertain parameter in the EPICYST sensitivity analysis [56]. The rate of human pork meal procurement was also considered a significant parameter in the EPICYST sensitivity analysis [56], so risk-factor analyses, such as those conducted in Western Kenya [83], could refine the uncertainty around this nominal parameter value in different settings. Fitting appropriate distributions such as the negative binomial distribution to *T*. *solium* cyst abundance data from pigs, could help to better determine the degree of infection aggregation, possibly indicative of heterogeneous exposure and support modelling overdispersion explicitly as performed for *E*. *multilocularis* worm burden in foxes [45].

### Population-based versus Individual-based modelling approaches

Transmission dynamics models of *Echinococcus* spp. reveal an interesting split between modelling approaches. Deterministic, population-based transmission models have been used primarily for *E*. *granulosus* incorporating dogs as the definitive host, sheep or other livestock as the intermediate host and humans acting as accidental intermediate hosts [49, 50, 54]. The exception is Huang et al. [48] where an individual-based model of *E*. *granulosus* was developed to study dynamics in a small community, an approach also applicable to the simulation of *T*. *solium* in small communities. Wang et al. [54] extends the models previously developed [49,50] by devising an approach to tackle parameter estimation issues concerning egg dynamics in the environment. The model incorporates infection delays as distributed time delays of infection between hosts, with different distribution functions chosen to reflect differences in the range of host movement (e.g., livestock, humans), and may provide an alternative approach for modelling transition between infection stages in the *T*. *solium* transmission models.

*E*. *multilocularis* transmission models were initially structured within deterministic, population-based frameworks [34, 38], also extending these approaches to consider optimal control through an economic lens [47]. Takumi & Van der Giessen, 2005 [40] also present an *E*. *multilocularis* deterministic model which tracks the mean number of transmission stages in hosts rather than measuring prevalence to replicate more accurately the rebound to pre-control of adult worm prevalence seen after cessation of a deworming campaign, even when substantial reductions are initially achieved. The impact on the rate at which average worm burdens return to pre-control levels, following cessation of community chemotherapy interventions has been further demonstrated for other helminths [84]. Modelling of *E*. *multilocularis* transmission dynamics diverges significantly from *E*. *granulosus* modelling through the development of individual-based stochastic dynamics [37, 44, 57] in the definitive host (e.g., foxes) to capture stochasticity in the demographic and infection processes in the wild animal populations that drive transmission. Heterogeneities in local pig populations and differential pig foraging behaviours [85] may be better captured by similar individual-based techniques, although these behaviours may be difficult to parameterise reliably and will likely be seasonally- and husbandry/management system-specific.

### Spatial and seasonal transmission modelling

Recognising that environmental contamination is spatially aggregated [40], *E*. *multilocularis* transmission models [37, 38, 45, 57] and a specific *T*. *ovis* deterministic transmission model [51] have introduced spatial dynamics by a variety of approaches (Table 5). Spatial heterogeneity in *T*. *solium* transmission is undoubtable and has been identified in a number of settings, with the detection of local clusters of pig cysticercosis prevalence and incidence [86,87], and clustering of pig cysticercosis infection (or seropositivity) near to human taeniosis carriers [88–90]. This may indicate the presence of spatially-aggregated environmental contamination of *T*. *solium* eggs and suggests spatially heterogeneous transmission. There is some evidence to suggest that other mechanisms are involved in the spatial distribution of *T*. *solium* eggs in the environment, such as the possible role of dung beetles acting as mechanical vectors for egg dispersal [91] and could be involved in a complex interplay with pig behaviour and seasonal factors [4]. Movement of individuals (humans and/or pigs) between communities may also play an important role in *T*. *solium* transmission and will influence the likelihood of sustaining elimination or experiencing resurgence [35]. Inclusion of spatial dynamics, however, should not detract from resolving the structural and parametric uncertainties that affect the current non-spatial models.

**Table 5:** Spatial modelling approaches (defined as incorporation of explicit spatial structure linked to transmission processes) used in in transmission models for wider Taeniidae family models.

Model & species	Approaches to spatial modelling
Hansen et al. 2003 [37] *Echinococcus multilocularis*	Grid-based: foxes are modelled as individual animals and voles as population units (in grids) in foxes’ territory (with foxes randomly distributed). Fox interaction (capture prey, defecate) is based on random draws per ‘territory’. Eggs shed in faeces are represented by position on grid–subpopulation of voles become infected if in infected grid during a time-step.
Milner-Gulland et al. 2004 [39] *E*. *multilocularis*	*E*. *multilocularis* cysts modelled as individuals in sedentary rodent population (hosts not modelled). Rodent population density and vegetation type (using GIS data) determines carrying capacity /habitat suitability of a patch. Density-dependence in intermediate host modelled as non-linear relationship between habitat suitability and carrying capacity & ‘scramble-type’ density- dependence. Dispersal of parasite through rodent consumption by foxes/movement of foxes and rate of release of eggs into a patch by adult worms (fecundity rate)–dependent on carrying capacity (or rodent availability–reflected by cyst population in a patch). A metapopulation is constructed of 9 patches representing the transitional area or marginal semi-arid area between wet steppe (high prevalence) and desert area (no infection). Annual time-step modelled.
Takumi et al. 2008 [45] *E*. *multilocularis*	The mean worm burden is modelled at a given time and location, incorporating parameters for exponential growth of the worm population and a diffusion coefficient (Km^2^ per year) to take into account the rate of spread of the parasite from an initial localised infection focus. The spatial model was fitted to spatial and longitudinal worm burden data in the border area of the Netherlands (with Germany and Belgium).
De Wolf et al. 2013 [51] *Taenia ovis*	Total pasture area is divided into equally-sized zones. Dog defecation at random in a zone becomes "hot" (equates to heavily contaminated). A model parameter is included to estimate rate of contact of susceptible lambs with "hot zones", defined as an area where susceptible sheep would be exposed to sufficient numbers of eggs (~ 100 eggs) to produce sufficient cysticerci to permit condemnation of carcasses and subsequent dog infection. Over time eggs disperse and decay (fixed- set to 12 weeks per zone).
Budgey et al., 2017 [57] *E*. *multilocularis*	Habitat is modelled as a 'mesh', with each cell representing 0.25 km^2^ & fox dens distributed randomly to match local densities from data, with foxes spending 90% of time in home territory (grid). Foxes exposed to a proportion of vole population that is infected in territory (vole dynamics modelled at population level). Defecation with infective material is distributed homogenously throughout territory. Egg survival times are dependent on temperature; viable egg numbers fall asymptotically in each territory. The total number of eggs in the environment dictates the infected proportion of susceptible voles.

GIS: Geographical information system.

Another feature explored in the *E*. *multilocularis* modelling papers is the impact of seasonal variation, by seasonal forcing of transmission models to account for differences in egg viability and movement of wild animal populations between seasons, for example by describing egg decay as a function of temperature [57]. The Gonzalez et al. [35] *T*. *solium* transmission model begins to consider the possibility that *T*. *solium* dynamics may be influenced by climate; however, there is little information available to estimate the effect of temperature (and other environmental variables) on *T*. *solium* egg viability in natural conditions. These factors might also affect transmission differently depending on the endemicity level, e.g. the proportions of infections in pigs resulting from indirect transmission. The role of peak pork consumption periods [92] could provide a more realistic way of implementing *T*. *solium* seasonal dynamics and would be interesting to explore with relevant longitudinal data. Advanced statistical modelling approaches have also been adopted in the wider *Echinococcus* modelling literature to improve predictive ability where periodicity in human echinococcosis prevalence data is observed [93]; however, fairly detailed time-series data are required for model fitting. Further seasonal heterogeneities may exist including seasonal slaughter patterns in areas where more pigs are slaughtered due to specific holidays [92], to obtain capital ahead of planting season, or the free capital for school fees. Likewise, seasonal variation in local crop production systems have a potential impact on transmission dynamics [3]. Less predictable events such as funerals can additionally lead to increased slaughter activity and movement of pigs.

### Data needs and future collaborations

**Fig 2:**
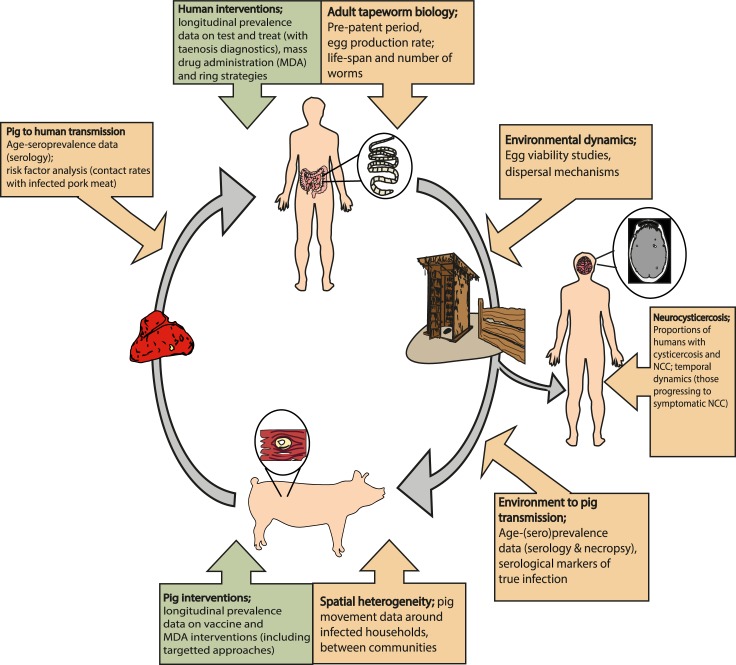
Identifying key research gaps and data needs towards a comprehensive research agenda for *Taenia solium* epidemiology, control and elimination. NCC- neurocysticercosis; MDA- Mass drug administration.

A number of data gaps are evident to inform modelling efforts and develop a comprehensive research agenda for *T*. *solium* control and elimination efforts, with Fig 2 summarising data needs described across this paper. It is clear that one of the limitations of existing *T*. *solium* transmission models is uncertainty surrounding biological parameter estimates, for example, for those associated with egg dynamics and the adult tapeworm lifespan, identified as influential parameters (egg production rate/ death rates) in the EPICYST sensitivity analysis [56]. Direct measurement is often difficult through experimental design, for example for egg production rates; therefore, it could be possible to use the existing or improved *T*. *solium* models to infer these values from observable data, such as fitting to baseline prevalence data. Transmission rate (FoI) parameterisation with FoI model fitting for different settings as applied for *E*. *granulosus* [36, 48] and *Trypanosoma cruzi* [94] could be facilitated with collection of detailed age-stratified prevalence and incidence data, using diagnostics with field-validated sensitivity and specificity estimates to perform suitable adjustments. Necropsy of pigs, which is the assumed gold standard diagnostic methodology, would provide the most robust and reliable data for model fitting; however, issues associated with cost and feasibility of obtaining reasonable sample sizes, longitudinal measurements, and utility in the control phase of a programme with low infection prevalence levels pose barriers to the use of these data. Determining serological diagnostic markers which represent true infection status will be important, as performed for validation of B158/B60 Ag-ELISA with necropsied animals in Zambia [95], to establish effectiveness of interventions where necropsy is unavailable.

Development of spatial transmission models, when the current uncertainty is addressed in existing models, will require spatially-resolved infection datasets, including variables on pig movement between communities and/or households, household georeferenced data, and data on human movement, as demonstrated for developing a spatially-explicit network model of endemic schistosomiasis in Senegal using mobile phone data [96].

Although not necessary for accurate transmission modelling, dynamic modelling of neurocysticercosis (NCC) to understand how interventions influence longer term burden of disease estimates would be useful for economic assessments. The main challenges associated with NCC modelling include simulating the proportions of individuals with cysticercosis that have neurocysticercosis, and the proportion subsequently developing morbidity and when this occurs (rather than those that are asymptomatic or presenting with mild symptoms), which would require temporal data [97]. Burden of disease modelling would also require data to capture the variation of infection-related morbidity. Clinical neurocysticercosis, for example, is highly pleomorphic, with a range of factors influencing clinical outcomes including the location of lesions within the central nervous system (e.g. extra- compared to intra- parenchymal), the cyst stage and the intensity of the immune response to cysts [98]. Bhattarai et al. [99] have included the DALYs for NCC associated headache in their burden of disease estimation, but more generally modelling efforts have focussed on morbidity associated with epilepsy and seizures. Relevant to transmission, the EPICYST model [56] also contains a compartment for humans infected with both cysticercosis and taeniosis, for which there are very limited data.

Finally, it is clear that simulated interventions need improved parameterisation in terms of efficacy and coverage and require longitudinal intervention datasets for validation. Reliable intervention modelling will require data on age-structured interventions, especially for pig-directed strategies such as vaccination and oxfendazole treatment (to model that animals close to slaughtering age should not be treated), but also for human-directed strategies such as school-based treatment programmes [77]. This type of intervention modelling is already implementable in cystiSim [53] and there are plans to integrate these interventions using an age-structured version of EPICYST [56]. A ‘logical model’ of pig cysticercosis infection risk in different age cohorts by Lightowlers & Donadeu [92] clearly outlines some of the considerations for an age-structured model. For example, the authors suggest restricting oxfendazole use in animals approaching the average age of slaughter, as oxfendazole treatment mandates a 21-day withholding period before human consumption. Equally, testing how the average age at which pigs are slaughtered impacts onward transmission risk and, therefore, intervention efficacy would be important to consider.

Working closely with field partners, stakeholders and strengthening collaboration between *T*. *solium* modelling groups will facilitate opportunities to harmonise models and compare projections through cross-validation based on longitudinal field data from intervention trials [100]. This approach will improve confidence in the predictive abilities and utility of *T*. *solium* transmission models for evaluating whether the WHO NTD roadmap targets, especially relating to the development of a validated strategy for control and elimination, will be achievable in the near future.

## Supporting information

S1 Table PRISMA checklist for systematic reviews. From: Moher D, Liberati A, Tetzlaff J, Altman DG, The PRISMA Group (2009). Preferred Reporting Items for Systematic Reviews and Meta-Analyses: The PRISMA Statement. PLoS Med 6(7): e1000097. doi: 10.1371/journal.pmed1000097.
(DOCX)

S1 Flow chart PRISMA Flow diagram detailing the number of studies screened, assessed for eligibility, and included in the Taeniidae family modelling review. (DOC)

S1 File Full data extraction tool: *T*. *solium* models and related transmission models of other Taeniidae family infections. Main sheet includes reference, motivation, model structural, parameterisation and intervention modelling details. Model assumptions and main findings included on separate sheets in file.
(XLSX)
