# Supplementary material for: Strategies for tackling Taenia solium taeniosis/cysticercosis: A systematic review and comparison of transmission models, including an assessment of the wider Taeniidae family transmission models
Source: PLoS Negl Trop Dis. 2019 Apr 10;13(4):e0007301. doi: 10.1371/journal.pntd.0007301 (PMC6476523; doi:10.1371/journal.pntd.0007301)
Supplement: S1 Table — From: Moher D, Liberati A, Tetzlaff J, Altman DG, The PRISMA Group (2009). Preferred Reporting Items for Systematic Reviews and Meta-Analyses: The PRISMA Statement. PLoS Med 6(7): e1000097. doi: 10.1371/journal.pmed1000097. (DOCX) [file pntd.0007301.s001.docx]

| **S1 Table.**  **PRISMA checklist for systematic reviews** | | | |  |
| --- | --- | --- | --- | --- |
| **Section/topic** | **#** | **Checklist item** | **Reported on page #** | **Additional information** |
| **TITLE** | | |  |  |
| Title | 1 | Identify the report as a systematic review, meta-analysis, or both. | 1 | We report the paper as a ‘systematic review’ in the title “Strategies for tackling Taenia solium taeniosis/cysticercosis: a systematic review and comparison of epidemiological models” |
| **ABSTRACT** | | |  |  |
| Structured summary | 2 | Provide a structured summary including, as applicable: background; objectives; data sources; study eligibility criteria, participants, and interventions; study appraisal and synthesis methods; results; limitations; conclusions and implications of key findings; systematic review registration number. | 2 | The abstract is structured in terms of background, methods/ principal findings - referring to the PRISMA checklist and results/ discussion and conclusions |
| **INTRODUCTION** | | |  |  |
| Rationale | 3 | Describe the rationale for the review in the context of what is already known. | 4 - 6 | This information is provided in the background |
| Objectives | 4 | Provide an explicit statement of questions being addressed with reference to participants, interventions, comparisons, outcomes, and study design (PICOS). | NA | Not applicable as addressing modelling literature |
| **METHODS** | | |  |  |
| Protocol and registration | 5 | Indicate if a review protocol exists, if and where it can be accessed (e.g., Web address), and, if available, provide registration information including registration number. | NA | Review protocol not available. |
| Eligibility criteria | 6 | Specify study characteristics (e.g., PICOS, length of follow-up) and report characteristics (e.g., years considered, language, publication status) used as criteria for eligibility, giving rationale. | 6 | Outlined in Methods: Search strategy and assessment and Selection criteria and assessment sections |
| Information sources | 7 | Describe all information sources (e.g., databases with dates of coverage, contact with study authors to identify additional studies) in the search and date last searched. | 6 | Outlined in Methods: Selection criteria and assessment section |
| Search | 8 | Present full electronic search strategy for at least one database, including any limits used, such that it could be repeated. | 6 | Outlined in Methods: Search strategy and assessment section |
| Study selection | 9 | State the process for selecting studies (i.e., screening, eligibility, included in systematic review, and, if applicable, included in the meta-analysis). | 6 | Outlined in Methods: Selection criteria and assessment section |
| Data collection process | 10 | Describe method of data extraction from reports (e.g., piloted forms, independently, in duplicate) and any processes for obtaining and confirming data from investigators. | 6 | Outlined in Methods: Search strategy and assessment section |
| Data items | 11 | List and define all variables for which data were sought (e.g., PICOS, funding sources) and any assumptions and simplifications made. | 6 | Outlined in Methods: Search strategy and assessment section |
| Risk of bias in individual studies | 12 | Describe methods used for assessing risk of bias of individual studies (including specification of whether this was done at the study or outcome level), and how this information is to be used in any data synthesis. | NA | Not applicable given review of modelling literature |
| Summary measures | 13 | State the principal summary measures (e.g., risk ratio, difference in means). | NA |  |
| Synthesis of results | 14 | Describe the methods of handling data and combining results of studies, if done, including measures of consistency (e.g., I^2^) for each meta-analysis. | 10 | This information is provided in the first paragraph of the Results and Discussion |

*From:*  Moher D, Liberati A, Tetzlaff J, Altman DG, The PRISMA Group (2009). Preferred Reporting Items for Systematic Reviews and Meta-Analyses: The PRISMA Statement. PLoS Med 6(7): e1000097. doi:10.1371/journal.pmed1000097

For more information, visit: **www.prisma-statement.org**.
