# Supplementary material for: Strategies for tackling Taenia solium taeniosis/cysticercosis: A systematic review and comparison of transmission models, including an assessment of the wider Taeniidae family transmission models
Source: PLoS Negl Trop Dis. 2019 Apr 10;13(4):e0007301. doi: 10.1371/journal.pntd.0007301 (PMC6476523; doi:10.1371/journal.pntd.0007301)
Supplement: S1 Flow chart — (DOC) [file pntd.0007301.s002.doc]

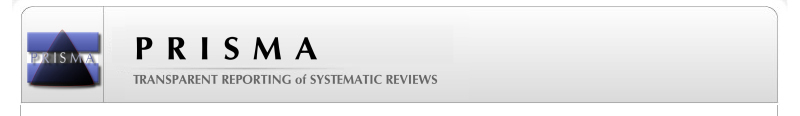
**PRISMA 2009 Flow Diagram**

**Screening**

**Included**

**Eligibility**

**Identification**

Records identified through database searching
(n = 944)

Additional records identified through other sources
n = 5 (known to authors (n = 2) & additional searches (n = 3))

Records screened
(n = 949)

Records excluded
(n = 899)

Full-text articles assessed for eligibility
(n = 50)

Full-text articles excluded, with reasons
(n = 22)

Studies included in qualitative synthesis
(n =28)
